# Determinants of vaccination uptake, and influenza vaccine effectiveness in preventing deaths and hospital admissions in the elderly population; Treviso, Italy, 2014/2015-2016/2017 seasons

**DOI:** 10.1080/21645515.2019.1661754

**Published:** 2019-10-07

**Authors:** Stefania Bellino, Cinzia Piovesan, Antonino Bella, Caterina Rizzo, Patrizio Pezzotti, Mauro Ramigni

**Affiliations:** a Department of Infectious Diseases, Italian National Institute of Health (Istituto Superiore di Sanità, ISS), Rome, Italy; b Department of Epidemiology, Local Health Unit 2 Marca Trevigiana, Treviso, Italy; c Direction of Clinical Departments, Bambino Gesù Children's Hospital, Rome, Italy

**Keywords:** Influenza, vaccine effectiveness, mortality rate, hospitalization rate, cohort study, Italy

## Abstract

Seasonal influenza is an important cause of morbidity and mortality, particularly among the elderly population. Determinants of vaccination uptake and its impact on health outcomes in the seasons 2014/2015–2016/2017 in elderly living in Treviso area (Veneto Region, North-Eastern Italy) were evaluated. A retrospective cohort study was conducted combining information from several health administrative databases, and multiple Poisson regression models were applied to evaluate the influenza vaccine effectiveness, also adjusting for confounding factors. MF59-adjuvanted trivalent-inactivated vaccine was mainly administered. Data from more than 83,000 elderly people were analyzed by year. Vaccine coverage was about 50%; influenza vaccination uptake was independently associated with older age, male sex, increasing number of underlying chronic conditions, previous pneumococcal vaccination, annual expenses for specialist medical cares, and general practitioner to whom the elderly was in charge. After adjusting for previously described characteristics, vaccination was associated with lower mortality and influenza-related hospitalization rates. Specifically, during influenza season the adjusted incidence rate ratio of death and of influenza-related hospitalizations for vaccinated compared to unvaccinated persons was 0.63 [95% confidence interval (CI) 0.58–0.69, *p* < .001] and 0.86 (95% CI 0.81–0.91, *p* < .001), respectively. A similar effectiveness was estimated for death in all age groups (≤74, 75–84, ≥85 years old), whereas a higher effect was found for hospitalizations in subjects aged ≥75 years old. Vaccination was also effective both in males and females. Findings suggest a health benefit of the influenza vaccination in the elderly population. Efforts should be focused on strategies to increase the vaccination uptake as important instrument of prevention.

## Introduction

Influenza is a serious public health problem and a significant source of direct and indirect costs for the implementation of control measures and management of cases and complications. Influenza virus results in annual epidemics, with 3–5 million cases of severe illness worldwide, and 290,000 to 650,000 respiratory deaths, most of them among elderly people, defined as those aged 65 years and older.^1^ Furthermore, elderly population, and more specifically those with chronic underlying conditions, are at increased risk for hospitalization due to influenza complications.^2^ Indeed, influenza may increase the severity of chronic lung diseases, and viral pneumonia due to influenza seems to predispose to myocardial infarction and congestive heart failures.^3^

Vaccination is currently the most effective measure to prevent influenza or to reduce its morbidity. However, the need of evidence on vaccine effectiveness, its impact, and value across target groups and among seasons continue to generate a large discussion.^4^ In addition, evidence of the effectiveness of flu vaccine in preventing severe clinical outcomes was recently described as low or very low among elderly people.^5^ As a consequence, despite the Council of the European Union and the World Health Organization (WHO) recommended to annually vaccinate elderly people,^6,7^ influenza vaccine coverage in this group remained below the 75%, target in most European countries.^8^ In Italy, a decreasing trend in the vaccine coverage was observed since 2008/09, and in the last seasons, it was around 50% in elderly people,^9^ despite the recommendation of the Italian Ministry of Health through the National Vaccination Prevention Plan and the recommendations for influenza prevention published every season.^10^

Another important aspect is that the vaccines developed to protect against seasonal influenza illness are updated every year in an effort to match the influenza A (B) vaccine strain with currently circulating viruses.^11^ Indeed, influenza viruses change rapidly due to antigenic drift so that vaccines are reformulated and delivered annually, commonly through seasonal campaigns. Licensed vaccines include inactivated or live-attenuated influenza A (B) strains, with three or four subtypes per vaccine.^12^ Since flu vaccines could be less effective in older than in younger adults, due to the weaker immune responses to vaccination for an age-related decline in immune function, strategies to enhance vaccine immunogenicity and overcome the limitations of immunosenescence include the use of high antigen doses and the formulation of vaccine with an adjuvant.^13^

Until now, only few studies were performed in Italy, aimed at assessing the effectiveness of influenza vaccination in reducing mortality and hospitalization due to pneumonia or influenza among elderly subjects, with heterogeneous results in terms of percentage of protection,^14–18^ or even no effectiveness.^19^

In this context, we performed a retrospective cohort study to evaluate the vaccination coverage and its determinants, as well as to estimate the influenza vaccine effectiveness (IVE) in preventing deaths and hospital admissions in the elderly population in a North-Eastern Italian Province of the Veneto Region, during 2014/2015-2016/2017 seasons.

## Methods

### Setting

The study refers to people living in the area of Treviso province (Treviso city, and other 36 municipalities, around 400,000 inhabitants (around 90,000 ≥ 65 years old), half of the entire province) in Veneto Region in the North-East of Italy. This is the area covered by the ex-Local Health Unit (LHU) 9 and now part of the LHU called ULSS (Unità Locale Socio Sanitaria) 2 – Marca Trevigiana. LHU is the territorial unit into which Italian National Health Service is divided. In 2017, LHUs in Veneto Region were reorganized, and now the ULSS 2, which covers the entire Treviso province, includes LHU 9 – Treviso, LHU 7 – Pieve di Soligo, and LHU 8 – Asolo.

### Influenza vaccination offer

Vaccination is offered free of charge to people aged 65 years or older, to other individuals aged 6-months–64 years with specific chronic conditions (such as diabetes, pulmonary and cardiovascular diseases, renal dysfunctions, cancer, immunodeficiency), to pregnant women, to people working in public services, and to healthcare workers. Vaccination campaigns were performed between November 3^rd^ and 28^th^ in 2014, November 9^th^ to December 4^th^ in 2015, and November 14^th^ to December 9^th^ in 2016. A single 0.5 mL dose of MF59-adjuvanted trivalent-inactivated vaccine was usually administered by general practitioners (GP) to their elderly patients. This vaccine has been recommended for adults 65 years and older by local health authority since the early 2000s. GPs are required to return the unused vaccines to the LHU the following week the date of the end of the campaign (28 November, 4 December, 9 December, in 2014, 2015 and 2016, respectively); therefore, all subjects were immunized within mid-december. Moreover, vaccinated subjects were identified by GPs who captured influenza vaccination data (vaccination date and type/brand of vaccine) through a process in which data were initially recorded on paper and subsequently manually entered into a database. However, GP did not always report the exact previous information on vaccination date and vaccine type/brand, therefore he may have used other non-adjuvanted trivalent/quadrivalent vaccines.

### Study design and data sources

We performed a retrospective cohort study combining information from several health administrative databases from the LHU 9 of the Veneto Region. More specifically, different health electronic databases were linked at the individual level: (1) the influenza vaccination registry, to evaluate whether the individual had been vaccinated before the influenza season start; (2) the Veneto vaccination database, to evaluate if the individual had received pneumococcal vaccination. Pneumococcal vaccination is administered in dedicated LHU vaccination centers (together with all other offered vaccinations except for influenza), and the information is recorded in the Regional Vaccination Registry; (3) the ACG database (Adjusted Clinical Groups, The Johns Hopkins ACG® System) containing information on comorbidities and healthcare resource utilization.^20,21^ In brief, for each individual registered in Veneto Health Care Population Registry (a compulsory registry for receiving universal coverage), data on diagnoses, drugs, procedures and costs were retrieved from the administrative healthcare databases routinely available in the Veneto Region (i.e., Hospital Discharge abstracts, Emergency Room visits, Chronic disease registry for co-payment exemptions, ambulatory visits and medications, and the Home care database). With regard to drug expenditure and usage, only drugs reimbursed by the Regional Health Service were considered, as over-the-counter drug data were not available. Costs were calculated on the basis of a medications’ actual costs and of inpatient/outpatients. The underlying chronic conditions were established using the EDC (Expanded Diagnosis Clusters), which coincides with clinical diagnoses that the ACG System assigns to single patients by combining different information ﬂows as described above; (4) the Regional Hospital Discharge Records, including also hospital admissions in other Italian Regions, to assess dates of hospital admissions, main and secondary clinical diagnoses of discharges and (5) the Mortality database, to obtain date of death. For the latter, specific causes of death were not available; therefore, only death for all-causes was considered as outcome. Moreover, it was not possible to collect information on the laboratory-confirmed influenza cases.

### Statistical analysis

All people aged ≥65 years old living in the municipalities of the LHU 9, alive at December 15^th^ of each year (2014-2015-2016) were included in the analysis. Residents in long-term care facilities or assigned to a GP out of LHU 9 were excluded from the analysis because it was not possible to establish if they were or not vaccinated for influenza. Analyses were performed both stratifying by season-year and pooling calendar years. Influenza vaccination coverage was calculated as the ratio between vaccinated and exposed elderly in the LHU at December 15^th^ of each year. Data were stratified by the following characteristics: sex, age groups (5-year interval before 90 and ≥90 years old), number of underlying chronic conditions (0, 1, 2, 3, >3), previous pneumococcal vaccination (yes/no), annual expenses for specialist medical care (<300, 300–700, 700–1,700, ≥1,700 Euro, roughly corresponding to the quartile values), and calendar year. Adjusted incidence rate ratios (IRR) of being vaccinated were estimated by a multi-level Poisson regression model,^22^ including all the above-mentioned variables. GP was included as a random effect first-level clustering variable; age and sex of the GP were initially included as fixed effect but, given that they were not significantly associated to the outcome, they were not included in the model here reported.

To evaluate the IVE, two outcome measures were considered: mortality rate (all-causes) and hospital admission rate (all-causes and influenza-related) that occurred between December 16, 2014, and December 14, 2017. IVE has been estimated as (1 – IRR) x 100, where IRR denotes the confounder-adjusted incidence rate ratio, comparing the death/hospitalization incidence rate among the vaccinated subjects to the death/hospitalization incidence rate among the unvaccinated subjects. IVE was evaluated in four different periods: December 15 to March 31 (Influenza season), April 1 to June 30 (post-influenza season), July 1 to September 30 (summer season), October 1 to December 14 (pre-influenza season). We considered this stratification in four periods to test the hypothesis that outside the influenza season no risk difference, in terms of deaths and influenza-related hospitalizations between vaccinated and unvaccinated individuals, should be detected. To define the influenza-related hospitalizations, the following ICD-9-CM (International Classification of Diseases, 9^th^ Revision, Clinical Modification) codes, recorded as primary or secondary diagnoses of discharge, were selected: 487 influenza; 480–486 pneumonia; 460–466, 490–496, 500–508, 510–516 respiratory diseases; 410, 422, 427, 428 cardiovascular diseases^23-25^ (a detailed description of the diagnoses codes has been reported in Supplementary Table S1).

We considered each hospitalization event unless the same patient experienced readmissions within 3 days after the previous discharge that were due to transfers between hospitals. These re-admissions, being a continuation of a single hospitalization event, were managed accordingly.

To estimate mortality and hospitalization IRR for vaccinated versus unvaccinated, univariable and multivariable Poisson regression models,^26^ using clustered sandwich estimator standard error that allows for intra-patient correlation, were applied, including the interaction between vaccination and season-year together with the previously described covariates. Although in the preliminary analyses we considered Poisson multilevel models with GP as a random effect, in the final models it was not included because it did not impact the parameter estimates. Regarding the risk of death, the “zero-inflated” Poisson model was applied,^27^ given that this provided a significant best fit, as suggested by the Vuong test,^28^ compared to the ordinary one. In addition, to evaluate if the estimated IVE on the mortality rates was different by age groups (i.e., <75, 75–84, ≥85 years old), sex, and pneumococcal vaccine administration, a “zero inflated” Poisson model with the interaction terms between flu vaccination and sex, age group, and pneumococcal vaccine was also considered, always adjusted for chronic underlying conditions and expenses for medical cares. Same models were applied also to the outcome rate of hospitalization for any cause, and rate of influenza-related hospitalizations; for both outcomes a standard Poisson model, instead of a “zero-inflated” one, was used, because the latter did not add any improvement in the goodness of fit. Finally, to take into account among the potential confounding factors and effect modifiers the influenza vaccination in any of the previous two seasons, a subgroup analysis was performed considering only subjects who contributed to all three cohorts. All models considered as exposure time for each subject the number of days of each period or until the date of death if the subject died before the end of the period, or until the date of change of residence if the person moved to a municipality outside the LHU 9 territory before the end of the period.

All analyses were performed using the Stata software, version 13 (Stata Cooperation, College Station, Texas, USA).

## Results

The study initially included three cohorts of elderly subjects of 86,724, 83,333, and 89,642 people at December 15 of 2014, 2015, and 2016, respectively. Residents in long-term facilities (2.5% in 2014, 2.4% in 2015, 3.0% in 2016) or assigned to a GP out of the LHU 9 (1.5% in 2014, 2.1% in 2015, 1.3% in 2016) were excluded from the analysis. Therefore, the evaluated people were 83,265, 83,375, and 85,800 at December 15 of 2014, 2015, and 2016, respectively (see Supplementary Figure S1). Overall, study participants were 93,492, who contributed to at least one cohort year. Specifically, 75,148, 9,652 and 8,692 contributed to three, two and one cohort year, respectively. Person-years during the study period were 125,253 for vaccinated, and 123,752 for unvaccinated.

### Circulating influenza strains and vaccination coverage

Table 1 shows a summary of the circulating strains overall in Italy and particularly among elderly people in the Veneto Region. In the 2014/15 season, a virus was predominant (84%), with the co-circulation of H1N1 (52%) and H3N2 (41%) subtypes, whereas during the following 2015/16 season, the co-circulation of A (43%; A/H1N1 35%, A/H3N2 56%) and B (57%; Victoria lineage 95%) viruses was observed; finally, in the 2016/17 season the A/H3N2 virus subtype predominated (93%). Regarding the elderly in Veneto Region, there were few differences with the viruses isolated in all population in Italy, except for 2015/16 season, in which a lower percentage of B virus (18%) was detected among elderly. Of note, during 2015/16 and 2016/17 seasons, the influenza B circulating strains at national level were antigenically distinct from those selected for the B component in the trivalent influenza vaccine.

**Table 1.** Circulating virus strains, characteristics of vaccines for influenza seasons, and influenza and pneumococcal vaccine coverage in the elderly, LHU 9, Treviso, Veneto Region, Italy, 2014–2017.

		Circulating viruses in Italy and Veneto Region		Influenza Vaccine coverage		Pneumococcal vaccine coverage		Pneumococcal vaccine type
Influenza Season	Composition of influenza virus vaccine	Italy (all population)	Veneto Region (only elderly)	Elderly, Italy	Elderly – LHU 9, Treviso (Veneto Region)	Elderly – LHU 9, Treviso		
2014-2015	A/California/7/2009 (H1N1) A/Texas/50/2012 (H3N2) B/Massachusetts/2/2012 (Yamagata lineage)	A 84% (H1N1 52%, H3N2 41%) B 16% (Yamagata 97%)	A 92% (H1N1 55%, H3N2 30%) B 8% (not subtyped)	48.6%	50.0% (53.4%)	15.3%	PPV23 (99.6%) PCV13 (0.2%) PCV7 (0.2%)	
2015-2016	A/California/7/2009 (H1N1) A/Switzerland/9715293/2013 (H3N2) B/Phuket/3073/2013 (Yamagata lineage)	A 43% (H1N1 35%, H3N2 56%) B 57% (Victoria 95%)	A 82% (H1N1 8%, H3N2 75%) B 18% (Victoria 74%)	49.9%	49.6% (54.0%)	15.8%	PPV23 (77.5%) PCV13 (22.4%) PCV7 (0.2%)	
2016-2017	A/California/7/2009 (H1N1) A/Hong Kong/4801/2014 (H3N2) B/Brisbane/60/2008 (Victoria lineage)	A 95% (H1N1 1%, H3N2 93%) B 5% (Yamagata 96%)	A 93% (H3N2 90%) B 7% (Yamagata 88%)	52.0%	51.6% (55.8%)	15.1%	PPV23 (54.0%) PCV13 (45.8%) PCV7 (0.2%)	

WHO recommendations on the composition of influenza virus vaccines, available at https://www.who.int/influenza/vaccines/virus/recommendations/en/.

Italian coverage, data available at: http://www.salute.gov.it/portale/influenza/dettaglioContenutiInfluenza.jsp?lingua=italiano&id=679&area=influenza&menu=vuoto.

Circulating viruses in Italy, InfluNet reports, available at: http://old.iss.it/fluv/index.php?lang=1&anno=2019&tipo=13.

PCV7, 7-valent pneumococcal conjugate vaccine; PCV13, 13-valent pneumococcal conjugate vaccine; PPV23, 23-valent pneumococcal polysaccharide vaccine.

LHU, Local Health Unit.

MF59-adjuvanted trivalent inactivated flu vaccine was mainly administered in the elderly living in the LHU 9, during the immunization campaigns although no data were available by type or brand of vaccine; the coverage was around 50% for each year, near to the national value. Pneumococcal vaccination was previously administered in nearly 15% of individuals (most received 23-valent polysaccharide vaccine).

Demographic and clinical characteristics at baseline (December 15^th^) of each calendar year for vaccinated/unvaccinated people with influenza vaccine are shown in Table 2. Influenza vaccinated subjects were, on average, older, had more chronic conditions and higher expenses for medical cares, but received less pneumococcal vaccination. In fact, in reference to the latter point, individuals who were not vaccinated with the pneumococcal vaccine received influenza vaccine more frequently than the others (52% vs 43%), particularly if conjugate vaccine PCV13 was not administered. Hypertension, congestive heart failure, metabolic disorders, and asthma were the most represented chronic diseases.

**Table 2.** Vaccination coverage, at December 15^th^, by demographic and clinical characteristics of the elderly residents in LHU 9, Treviso, Veneto Region, Italy 2014–2016.

	December 15, 2014						December 15, 2015						December 15, 2016					
	Vaccinated		Unvaccinated		Total		Vaccinated		Unvaccinated		Total		Vaccinated		Unvaccinated		Total	
	n	%	n	%	n	%	n	%	n	%	n	%	n	%	n	%	n	%
**Participants**	41,614	50.0	41,651	50.0	83,265	100.0	41,857	49.6	42,518	50.4	84,375	100.0	44,250	51.6	41,550	48.4	85,800	100.0
**Sex**																		
Female	23,265	49.5	23,746	50.5	47,011	56.5	23,237	48.9	24,239	51.1	47,476	56.3	24,293	50.6	23,746	49.4	48,039	56.0
Male	18,349	50.6	17,905	49.4	36,254	43.5	18,620	50.5	18,279	49.5	36,899	43.7	19,957	52.9	17,804	47.1	37,761	44.0
**Age group (years)**																		
65-69	7,096	30.7	16,002	69.3	23,098	27.7	7,406	30.9	16,587	69.1	23,993	28.4	7,871	33.9	15,329	66.1	23,200	27.0
70-74	9,339	47.1	10,507	52.9	19,846	23.8	8,833	46.6	10,111	53.4	18,944	22.5	9,708	47.9	10,580	52.1	20,288	23.6
75-79	10,000	58.4	7,117	41.6	17,117	20.6	10,200	57.7	7,481	42.3	17,681	21.0	10,662	59.2	7,362	40.8	18,024	21.0
80-84	7,649	64.5	4,212	35.5	11,861	14.2	7,665	63.5	4,402	36.5	12,067	14.3	8,074	64.8	4,378	35.2	12,452	14.5
85-89	4,923	66.0	2,532	34.0	7,455	9.0	5,077	66.5	2,552	33.5	7,629	9.0	5,193	67.2	2,538	32.8	7,731	9.0
≥90	2,607	67.1	1,281	32.9	3,888	4.7	2,676	65.9	1,385	34.1	4,061	4.8	2,742	66.8	1,363	33.2	4,105	4.8
**N. of Clinical conditions**																		
0	4,999	30.3	11,479	69.7	16,478	19.8	5,142	29.8	12,142	70.3	17,284	20.5	5,310	30.8	11,936	69.2	17,246	20.1
1	11,545	45.9	13,613	54.1	25,158	30.2	11,605	45.5	13,899	54.5	25,504	30.2	12,259	47.5	13,534	52.5	25,793	30.1
2	11,134	54.8	9,185	45.2	20,319	24.4	11,277	55.4	9,063	44.6	20,340	24.1	11,895	57.5	8,798	42.5	20,693	24.1
3	7,836	62.8	4,643	37.2	12,479	15.0	7,769	62.4	4,677	37.6	12,446	14.8	8,332	64.3	4,623	35.7	12,955	15.1
>3	6,100	69.1	2,731	30.9	8,831	10.6	6,064	68.9	2,737	31.1	8,801	10.4	6,454	70.8	2,659	29.2	9,113	10.6
**Clinical conditions**																		
Congestive heart failure	8,549	66.7	4,260	33.3	12,809	15.4	8,282	65.9	4,282	34.1	12,564	14.9	8,753	68.3	4,070	31.7	12,823	15.0
Ischemic heart disease	4,341	65.4	2,301	34.6	6,642	8.0	4,370	64.5	2,402	35.5	6,772	8.0	4,669	66.6	2,339	33.4	7,008	8.2
Hypertension	31,995	56.2	24,949	43.8	56,904	68.3	31,984	56.0	25,136	44.0	57,120	67.7	33,838	57.9	24,577	42.1	58,415	68.1
Metabolic disorders	16,858	58.8	11,822	41.2	28,680	34.4	17,019	59.2	11,745	40.8	28,764	34.1	18,646	61.4	11,720	38.6	30,366	35.4
Diabetes mellitus	7,961	61.9	4,896	38.1	12,857	15.4	7,935	61.1	5,060	38.9	12,995	15.4	8,396	63.2	7,890	36.8	13,286	15.5
COPD	1,294	68.5	595	31.5	1,889	2.3	1,309	70.4	550	29.6	1,859	2.2	1,287	71.1	523	28.9	1,810	2.1
Asthma	6,902	61.1	4,402	38.9	11,304	13.6	6,993	61.7	4,349	38.3	11,342	13.4	7,163	63.8	4,065	36.2	11,228	13.1
Immunodeficiency	129	52.0	119	48.0	248	0.3	28	40.0	42	60.0	70	0.1	41	56.9	31	43.1	72	0.1
Rheumatoid arthritis	393	51.7	367	48.3	760	0.9	406	52.5	368	47.5	774	0.9	454	52.8	405	47.2	859	1.0
Renal failure	1,134	59.5	773	40.5	1,907	2.3	1,122	60.1	744	39.9	1,866	2.2	1,185	62.7	704	37.3	1,889	2.2
Transplant	75	38.3	121	61.7	196	0.2	77	42.1	106	57.9	183	0.2	92	48.7	97	51.3	189	0.2
**Pneumococcal vaccination**	5,212	40.9	7,547	59.1	12,759	15.3	5,573	41.8	7,755	58.2	13,328	15.8	5,939	45.9	7,011	54.1	12,950	15.1
**Expenses for specialist medical care (Euros)**																		
<300	6,807	31.3	14,934	68.7	21,741	26.1	7,020	31.1	15,532	68.9	22,552	26.7	7,977	33.6	15,775	66.4	23,752	27.7
300-700	10,271	49.9	10,299	50.1	20,570	24.7	10,559	49.9	10,592	50.1	21,151	25.1	10,870	52.5	9,819	47.5	20,689	24.1
700-1,700	12,398	59.7	8,364	40.3	20,762	24.9	12,192	59.9	8,174	40.1	20,366	24.1	12,518	61.7	7,757	38.3	20,275	23.6
≥1,700	12,138	60.1	8,054	39.9	20,192	24.3	12,086	59.5	8,220	40.5	20,306	24.1	12,885	61.1	8,199	38.9	21,084	24.6

COPD, chronic obstructive pulmonary disease. Immunodeficiency: conditions that suppress the immune function due to underlying disease and/or therapy, i.e., people receiving chemotherapy, HIV infection. Expenses for specialist medical care: total amount spent in the 12 months before the start of the vaccination campaign. Percentages refer to the row total, except for Total column that refers to column total.

Considering only patients who contributed to all three-cohort years (75,148), 38.2% did not receive any vaccination, 40.9% were vaccinated in all seasons, 11.2% were vaccinated during two seasons, and 9.7% were vaccinated only 1 year. In particular, 89.9% of vaccinated subjects in 2016 received influenza vaccination in at least one of previous two seasons (74.9% received vaccinations in both previous seasons), whereas 84.0% of unvaccinated subjects in 2016 were not previously immunized (data not shown in table).

Both crude and adjusted IRR of being vaccinated were associated with demographic (sex, age groups) and clinical characteristics (number of chronic underlying conditions, previous pneumococcal vaccination, expenses for specialist medical care) (Supplementary Table S2). Specifically, in the multivariable model, the adjusted IRR was higher for males compared to females and for those who received pneumococcal vaccination compared to unvaccinated ones; IRR increased with age, with the number of chronic diseases, and with annual individual health costs. Also GP, as a random effect, was significantly associated with vaccination, and the percentage of vaccinated patients largely varied among them (Supplementary Figure S2). In the model including all three calendar years, an adjusted slightly higher IRR to be vaccinated was detected in 2016/17 as compared to 2014/15 (Supplementary Table S3).

### Risk of death

**Figure 1.**
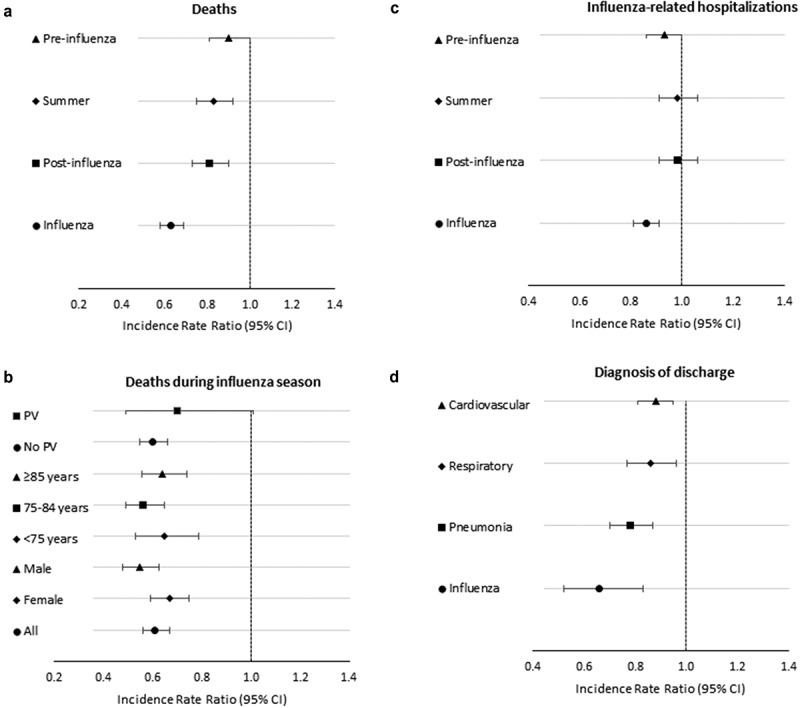
Adjusted mortality and hospitalization incidence rate ratios of vaccinated compared to unvaccinated elderly subjects, resident in Treviso Province, Veneto Region, Italy, during 2014/15, 2015/16, and-2016/17 seasons. Panel A: adjusted mortality rate ratios by season; panel B: adjusted mortality rate ratios by sex, age groups, and pneumococcal vaccination during influenza seasons; panel C: adjusted influenza-related hospitalization incidence rate ratios by season; panel D: adjusted hospitalization rate ratios by diagnosis of discharge during influenza seasons.

There were 8,131 deaths during the study period, 4,855 and 3,276 in vaccinated and unvaccinated subjects, respectively (Supplementary Table S4). Among deaths, the median age was 84 years, and 92% of them had at least one chronic disease. In the unadjusted analysis, vaccinated subjects had significantly higher mortality rates, both during influenza and non-influenza seasons; when adjusting the model for the other confounding factors, vaccination was associated with significant reductions in death (Table 3). Specifically, vaccination had significant estimated reduction of risk of death by 33%, 37%, and 39% (*p* < .001) during influenza season of 2014/15, 2015/16, and 2016/17, respectively, whereas during the post-influenza season reduction estimates ranged from 17% to 21% (*p* < .05). Only in the first year, a significant vaccination effect was observed also during summer. Regarding the other characteristics, adjusted IRR of death >1 were detected among males, older individuals, subjects with more chronic conditions and with greater annual expenses for medical cares, while a lower adjusted IRR [0.85, 95% confidence interval (CI) 0.74–0.98, *p* = .028] was found among pneumococcal vaccine recipients. In the pooled analysis made for all years, the adjusted IRR of death in vaccinated as compared to unvaccinated was lower during influenza period (IRR 0.63, 95% CI 0.58–0.69, *p* < .001) and increased progressively in the other non-influenza periods [IRR ranged from 0.81 (*p* < .001) to 0.90 (*p* = .054)] (Figure 1A). Considering only the influenza period, when including an interaction term between influenza vaccination administration (yes/no) and sex (male/female), age groups (≤74, 75–84, ≥85 years old) and pneumococcal vaccination (yes/no) we found that vaccination, having adjusted for all the previously described characteristics, had similar protective effect in all age groups and in vaccinated/unvaccinated people with pneumococcal vaccine (Figure 1B). Regarding sex, although in both groups influenza vaccination was estimated to be protective for the risk of death, the magnitude of the effectiveness was significantly different (IRR = 0.55, 95% CI 0.48–0.63 in males vs 0.67 95% CI 0.59–0.75 in females, *p* = .028).

**Table 3.** Crude and adjusted incidence rate ratios of death by season in LHU 9, Treviso, Veneto Region, Italy 2014–2017.

	Univariable analysis				Multivariable analysis			
	Crude IRR	95% CI		*p-value*	Adjusted IRR	95% CI		*p-value*
**Vaccinated vs Unvaccinated**								
***2014-2015***								
Influenza	1.30	1.14	1.48	*<0.001*	0.67	0.58	0.77	*<0.001*
Post-influenza	1.57	1.33	1.84	*<0.001*	0.81	0.69	0.97	*0.020*
Summer	1.44	1.23	1.69	*<0.001*	0.74	0.62	0.87	*<0.001*
Pre-influenza	1.63	1.38	1.94	*<0.001*	0.85	0.71	1.02	*0.087*
***2015-2016***								
Influenza	1.20	1.06	1.37	*0.005*	0.63	0.55	0.72	*<0.001*
Post-influenza	1.53	1.30	1.80	*<0.001*	0.79	0.66	0.94	*0.009*
Summer	1.66	1.42	1.94	*<0.001*	0.87	0.73	1.03	*0.111*
Pre-influenza	1.75	1.47	2.07	*<0.001*	0.92	0.77	1.11	*0.379*
***2016-2017***								
Influenza	1.17	1.02	1.34	*0.021*	0.61	0.53	0.71	*<0.001*
Post-influenza	1.55	1.32	1.82	*<0.001*	0.83	0.70	0.98	*0.031*
Summer	1.67	1.41	1.98	*<0.001*	0.91	0.76	1.09	*0.290*
Pre-influenza	1.72	1.44	2.06	*<0.001*	0.93	0.77	1.13	*0.468*
**Sex**								
Female	1.00				1.00			
Male	1.20	1.15	1.26	*<0.001*	1.49	1.42	1.56	*<0.001*
**Age group (years)**								
65-69	1.00				1.00			
70-74	1.51	1.36	1.69	*<0.001*	1.26	1.10	1.43	*<0.001*
75-79	2.83	2.57	3.12	*<0.001*	2.15	1.89	2.43	*<0.001*
80-84	5.40	4.91	5.93	*<0.001*	3.95	3.50	4.47	*<0.001*
85-89	10.27	9.36	11.26	*<0.001*	8.27	7.31	9.36	*<0.001*
≥90	21.87	19.96	23.96	*<0.001*	21.11	18.59	23.97	*<0.001*
**N. of chronic underlying conditions**								
0	1.00				1.00			
1	1.57	1.43	1.72	*<0.001*	1.12	1.01	1.23	*0.030*
2	2.59	2.37	2.83	*<0.001*	1.42	1.29	1.58	*<0.001*
3	3.71	3.39	4.07	*<0.001*	1.66	1.49	1.84	*<0.001*
>3	7.44	6.81	8.12	*<0.001*	2.37	2.13	2.64	*<0.001*
**Pneumococcal vaccination**	0.23	0.21	0.26	*<0.001*	0.85	0.74	0.98	*0.028*
**Expenses for specialist medical care (Euros)**								
<300	1.00				1.00			
300-700	1.30	1.19	1.42	*<0.001*	0.98	0.90	1.08	*0.743*
700-1,700	1.85	1.71	2.01	*<0.001*	1.16	1.06	1.28	*0.002*
≥1,700	5.91	5.50	6.35	*<0.001*	3.49	3.19	3.81	*<0.001*

Expenses for specialist medical care: total amount spent in the 12 months before the start of the vaccination campaign. CI, confidence interval. Periods were defined as follows: influenza, December 15– March 31; post-influenza, April 1– June 30; summer, July 1– September 30; pre-influenza, October 1– December 14. IRRs (incidence rate ratios) were obtained from a zero-inflated Poisson regression model.

### Risk of hospitalization

Hospital admissions for all-causes were more frequent among vaccinated (32,612) as compared to the unvaccinated subjects (23,714), as well as for those influenza-related (11,712 versus 6,643) (Table S4).

Crude hospitalization (at least one per period/year) rates were higher among vaccinated individuals (Table 4). When adjusting for possible confounding factors, vaccination was associated with a 12-16% reduction (*p* < .01) in the influenza-related hospital admission rates during the influenza season (Table 4). Conversely, no effect was found in pre- and post-influenza season as well as during summer (except in pre-influenza period during 2014–2015 season). Similarly, to death analysis, being male, older, with more chronic conditions, and greater expenses for medical cares were all conditions significantly associated with an increased risk of hospital admission, while a lower risk was found among pneumococcal vaccine recipients (Table 4).

**Table 4.** Risk of influenza-related hospital admission in the elderly by season in LHU 9, Treviso, Veneto Region, Italy 2014–2017.

	Univariable analysis				Multivariable analysis			
	IRR	95% CI		*p-value*	IRR	95% CI		*p*-value
**Vaccinated vs Unvaccinated**								
***2014-2015***								
Influenza	1.67	1.5139	1.84	*<0.001*	0.88	0.80	0.97	0.009
Post-influenza	1.93	1.71	2.18	*<0.001*	1.01	0.90	1.14	*0.899*
Summer	1.71	1.51	1.95	*<0.001*	0.90	0.79	1.03	*0.118*
Pre-influenza	1.65	1.45	1.88	*<0.001*	0.87	0.77	0.99	*0.036*
***2015-2016***								
Influenza	1.59	1.44	1.75	*<0.001*	0.84	0.76	0.93	*0.001*
Post-influenza	1.94	1.71	2.21	*<0.001*	1.02	0.90	1.16	*0.722*
Summer	1.98	1.74	2.26	*<0.001*	1.04	0.92	1.19	*0.518*
Pre-influenza	1.93	1.69	2.21	*<0.001*	1.02	0.89	1.16	*0.789*
***2016-2017***								
Influenza	1.58	1.43	1.75	*<0.001*	0.85	0.77	0.94	*0.002*
Post-influenza	1.71	1.51	1.94	*<0.001*	0.92	0.81	1.04	*0.191*
Summer	1.88	1.64	2.15	*<0.001*	1.02	0.88	1.16	*0.864*
Pre-influenza	1.67	1.46	1.90	*<0.001*	0.90	0.79	1.02	*0.111*
**Sex**								
Female	1.00				1.00			
Male	1.33	1.27	1.39	*<0.001*	1.39	1.34	1.45	*<0.001*
**Age group (years)**								
65-69	1.00				1.00			
70-74	1.71	1.55	1.88	*<0.001*	1.41	1.25	1.59	*<0.001*
75-79	3.09	2.82	3.38	*<0.001*	2.20	1.95	2.47	*<0.001*
80-84	5.34	4.90	5.84	*<0.001*	3.48	3.10	3.90	*<0.001*
85-89	8.18	7.49	8.92	*<0.001*	5.43	4.84	6.10	*<0.001*
≥90	11.85	10.81	12.99	*<0.001*	8.33	7.39	9.39	*<0.001*
**Number of clinical conditions**								
0	1.00				1.00			
1	1.67	1.53	1.81	*<0.001*	1.23	1.13	1.34	*<0.001*
2	2.95	2.71	3.20	*<0.001*	1.72	1.57	1.87	*<0.001*
3	4.86	4.47	5.28	*<0.001*	2.30	2.11	2.52	*<0.001*
>3	12.53	11.56	13.59	*<0.001*	4.30	3.92	4.71	*<0.001*
**Pneumococcal vaccination**	0.30	0.27	0.33	*<0.001*	0.95	0.83	1.09	0.476
**Expenses for specialist medical care (Euros)**								
<300	1.00				1.00			
300-700	1.49	1.38	1.61	*<0.001*	1.01	0.93	1.09	0.796
700-1,700	2.79	2.61	2.99	*<0.001*	1.39	1.28	1.49	*<0.001*
≥1,700	7.19	6.74	7.67	*<0.001*	2.85	2.66	3.07	*<0.001*

Expenses for specialist medical care: total amount spent in the 12 months before the start of the vaccination campaign. CI, Confidence Interval. Periods were defined as follows: influenza, December 15–March 31; post-influenza, April 1– June 30; summer, July 1–September 30; pre-influenza, October 1– December 14. IRRs (incidence rate ratios) were obtained from a Poisson regression model.

Pooling all calendar years, the IRR of influenza-related hospitalization of vaccinated versus unvaccinated during influenza period was 0.86 (95% CI 0.81–0.91, *p* < .001) and nearly 1 in the other periods (Figure 1C). When performing further multivariable Poisson models, including interaction terms between flu vaccination and sex, age-group, and pneumococcal vaccination, we found that there was a significantly different IRR for influenza vaccination within age groups: 1.00 (95% CI 0.88–1.14, *p* = .971) for people aged ≤74 years, 0.85 (95% CI 0.78–0.93, *p* < .001) for subjects aged 75–84 years, and 0.79 (95% CI 0.72–0.87, *p* < .001) for elderly ≥85 years; differences were detected between who received (IRR 1.24, 95% CI 0.96–1.60, *p* = .101) and not received (0.65, 95% CI 0.53–0.80, *p* < .001) pneumococcal vaccination.

Lower risks of hospitalization in vaccinated subjects were detected for all identified influenza-related causes during influenza season (Table 5). Specifically, vaccination was associated with 34% reduction in hospitalization rate for influenza (IRR 0.66 95%CI 0.52–0.83, *p* < .001), 22% for pneumonia (IRR 0.78 95%CI 0.70–0.87, *p* < .001), 14% for respiratory causes (IRR 0.86 95%CI 0.77–0.96, *p* = .006), and 12% for cardiovascular diseases (IRR 0.88 95%CI 0.81–0.95, *p* = .001) (Figure 1D).

**Table 5.** Risk of hospitalization in the elderly by diagnosis of discharge in LHU 9, Treviso, Veneto Region, Italy 2014–2017.

Outcome	Study period 2014–2017	Univariable analysis				Multivariable analysis			
Hospitalizations	Vaccinated vs unvaccinated	IRR	95% CI		*p-value*	IRR	95% CI		*p*-value
All causes	Influenza season	1.27	1.22	1.32	*<0.001*	0.87	0.85	0.90	*<0.001*
	Non-influenza season	1.40	1.36	1.44	*<0.001*	0.95	0.92	0.97	*<0.001*
Influenza-related	Influenza season	1.61	1.52	1.71	*<0.001*	0.86	0.81	0.91	*<0.001*
	Non-influenza season	1.82	1.73	1.91	*<0.001*	0.97	0.92	1.02	*0.173*
Influenza	Influenza season	1.14	0.91	1.43	*0.244*	0.66	0.52	0.83	*<0.001*
	Non-influenza season	1.21	0.50	2.92	*0.673*	0.70	0.29	1.69	*0.425*
Pneumonia	Influenza season	1.53	1.39	1.69	*<0.001*	0.80	0.72	0.88	*<0.001*
	Non-influenza season	1.82	1.67	1.98	*<0.001*	0.95	0.87	1.03	*0.199*
Respiratory diseases	Influenza season	1.71	1.54	1.90	*<0.001*	0.87	0.78	0.97	*0.014*
	Non-influenza season	2.01	1.82	2.22	*<0.001*	1.02	0.92	1.13	*0.657*
Cardiovascular diseases	Influenza season	1.74	1.61	1.87	*<0.001*	0.90	0.84	0.97	*0.007*
	Non-influenza season	1.85	1.74	1.96	*<0.001*	0.96	0.90	1.02	*0.168*

Periods were defined as follows: influenza season, December 15^th^ – March 31^st^, non-influenza season, April 1– December 14. IRRs (incidence rate ratios) were obtained from a Poisson regression model, adjusted for sex, age group, pneumococcal vaccination, number of chronic underlying conditions and expenses for specialist medical care.

### Subgroup analysis

Subgroup analysis, which considered only subjects who contributed to all three cohorts, highlighted that the influenza vaccination in any of the previous two seasons lowered both the mortality and influenza-related hospitalization rates in vaccinated as compared to unvaccinated subjects (Supplementary Table S5). Specifically, during influenza season 2016/17 the flu vaccination was associated with 66% reduction in mortality rates (IRR 0.34 95%CI 0.27–0.43, *p* < .001) in previously immunized subjects, whereas no effect was found in individuals who did not receive any vaccine in the previous 2 years (adjusted IRR 0.95 95%CI 0.65–1.39, *p* = .781). Similarly, vaccination was associated with 33% reduction in influenza-related hospitalization rates (adjusted IRR 0.67 95%CI 0.57–0.79, *p* < .001) in previously immunized subjects, while a not significant effect was observed in patients with no previously vaccination.

## Discussion

The present retrospective cohort study was aimed at evaluating vaccination coverage and vaccine effectiveness in preventing all-cause deaths and hospital admissions attributable to influenza in the elderly population living in an area of North-Eastern Italy, in the 2014/2015-2016/2017 seasons. Vaccination coverage in this area was around 50% in all three seasons, a value very close to the national one (see Table 1). In the multivariable analysis evaluating possible determinants of receiving influenza vaccination, we found that the IRR of vaccine uptake was significantly higher in males than in females, in older people, in people with chronic conditions, with higher annual expenses for specialist medical cares (considered as a further proxy of health status), and who received pneumococcal vaccination. Of note, in the univariable model, pneumococcal vaccination was associated with a lower risk to receive the influenza vaccination; indeed, subjects immunized with anti-pneumococcal vaccine were younger and with less co-morbidity as compared to those who were not immunized. Therefore, after adjusting for confounding variables the risk to be vaccinated was higher in those who received pneumococcal vaccination compared to unvaccinated ones.

Furthermore, although there was a statistically significant increase of the adjusted IRR in 2016/2017 compared to 2014/2015 the magnitude of increase was very small (IRR 1.03) and not really significant in terms of public health impact. There was also a significant and independently effect of the GP in the vaccination coverage, although no age and sex of the GP was associated with the vaccine uptake. These results are consistent with other studies conducted in other countries.^29–31^ Moreover, our data showed that influenza vaccination was usually received by the same individuals who were vaccinated in the previous seasons. This is in line with a recent systematic review on factors influencing flu vaccination behavior, which include previous vaccination history as one of the key factors, together with age, poor health status, medical service use, receiving knowledge/information from healthcare professionals.^32^

Regarding the effectiveness on reducing the risk of death (any cause) and the risk of influenza- related hospitalization, we found that, adjusting simultaneously for other individual characteristics, influenza vaccination was significantly associated with lower mortality and influenza-related hospitalization rates, and the benefit was found for all the discharge diagnoses including, influenza, pneumonia, respiratory, and cardiovascular diseases. Moreover, repeated influenza vaccination in the previous seasons influenced positively the vaccine effectiveness in the elderly. This is in line with recent published cohort studies in Stockholm County, Sweden^33^ that used similar outcomes as those reported in our study, and with a test negative design conducted in Valencia^34^ where the outcome was represented by confirmed influenza cases. In both studies,^33,34^ as well as in a recent meta-analysis,^35^ no negative effects of repeated seasonal vaccination were seen across more seasons, which strengthens the recommendation that persons belonging to this age group should be vaccinated yearly. Male sex, older age, more chronic conditions, and greater annual expenses for medical cares were significantly associated with an increased risk for both outcomes (death and hospitalization), while a lower risk was found among pneumococcal vaccine recipients. Of note, after adjusting for confounding factors, vaccination was associated with a reduction in the hospital admission rates only during the influenza season, whereas a lower, but still present, reduction in risk of all-cause mortality was found also in noninfluenza periods, suggesting that additional frailty indicators (*i.e*., severity of chronic conditions, perceived health status, socio-economic status, behavior-related factors) could be necessary to eliminate possible unknown biasing factors; however, these are not usually included in standard administrative healthcare databases. In the absence of phase III trials using clinical endpoints, non-experimental designs to evaluate vaccine effects are often used, although methods to reduce potentially confounding factors should be considered.^36^ Indeed, according to the European Medicines Agency Guideline on influenza vaccine,^37^ secondary outcomes should address the ability of vaccines to prevent pneumonia and influenza-related hospitalization (associated with respiratory or cardiac disease) as well as all cause of deaths. Therefore, in order to control for differences in health status and health-seeking behavior in vaccinated compared with unvaccinated individuals, information on potentially confounding factors has been collected, including chronic diseases, pneumococcal vaccination, and healthcare utilization indicated by the expenses for specialist medical care. We were able to collect the most important known confounding factors except for the severity of chronic diseases, socioeconomic status, and antiviral drug use. Moreover, negative confounding may occur, as high-risk groups are more likely to be vaccinated and therefore reduce IVE. Conversely, positive confounding may occur as a result of a ‘healthy vaccine effect’.^38^ People with a healthy lifestyle are more likely to accept/request vaccination, thus leading to an increase of measured IVE. Vaccine coverage has been found to be low in frail elderly patients; consequently, there may be relatively fewer severely ill patients in the exposed.^39–41^ Therefore, adjusting for negative and positive confounding factors through multivariable analysis should minimize this potential bias, to make sure that the observed differences in the occurrence of selected outcomes in vaccinated and unvaccinated subjects were due to the effect of the vaccine and did not reflect baseline differences between the two groups.

During the study different viruses circulated. In the 2014/15 period, A/H1N1 and A/H3N2 co-circulated and the season were characterized by a very high incidence of influenza-like-illnesses (ILI) and Intensive Care Unit (ICU) confirmed cases, especially in ≥65 years old individuals, with a mismatch between the A/H3N2 circulating virus and that included in the seasonal vaccine formulation.^42,43^ In Veneto Region, among severe acute respiratory infections (SARI) cases, A/H3N2 was predominant.^44^ During the following 2015/16 season, the co-circulation of A and B viruses was observed with a lower IVE estimate for influenza A(H1N1)pdm09, with respect to previous season due to the emerging genetic variant 6B.1.^45,46^ In Veneto Region, most ILI and SARI cases were infected with A/H3N2 virus.^47^ Moreover, during this season, the antigenic match between circulating strains (Victoria lineage) and vaccine composition (Yamagata lineage) did not fit for B. However, the detection rate of influenza type B virus among elderly in Veneto Region was low, in line with previous studies.^48^ In 2016/17 period, A/H3N2 virus predominated, and the season was characterized by a very high incidence of ILI and ICU confirmed cases, due to differences in glycosylation between A/H3N2 egg-adapted vaccines and circulating strains.^49–51^

Many previous studies examined IVE by analyzing a single or more seasons,^14–19,23^ with heterogeneous results. Recent observational studies in elderly adults have estimated that the adjuvanted vaccine provided significant protection against influenza-related hospitalization and that may further reduce influenza and pneumonia hospitalizations by an estimated 25% above those prevented by nonadjuvanted vaccine.^52^ In a study conducted in another north-eastern Italian area during 2016/17 season, it was found no significant effect of influenza vaccination in the elderly on the likelihood of hospitalization or death from pneumonia and influenza, indicating that some residual confounding might exist.^19^ However, another study conducted during 2014/15 season in the same Italian Region of the previous study showed that the overall risk of death was 18% lower among vaccinated compared to unvaccinated subjects (21% for pneumonia and influenza).^18^ A recent published test negative case–control study conducted in Italy during the 2016/17 season, to estimate vaccine effectiveness in preventing severe influenza in hospitalized elderly,^53^ showed a good adjusted IVE of the MF59-adjuvated vaccine against A(H1N1)pdm09 strains and a moderate adjusted IVE against B strains.

A study in US showed that vaccination in people with type 2 diabetes was associated with lower admission rates for pneumonia or influenza (IRR 0.85), and acute cardiovascular events (IRR 0.70 for stroke, 0.78 for heart failure).^23^ In a study conducted in New Zealand from 2012 to 2015, influenza vaccination was associated with a 59% reduction in the risk of influenza-associated intensive care unit admissions among hospitalized influenza confirmed cases.^54^ Finally, a multicentre case–control study conducted in 11 European countries estimated that in the 2015–16 influenza season vaccination prevented approximately half of the hospitalized laboratory-confirmed influenza cases among the elderly population.^55^

In interpreting our results, we need to consider some limitations. Relating to the representativeness of the study population and thus the generalizability of results, the study covered a part of a North-East Region of Italy and not the entire country. Vaccinated subjects were identified only by general practitioners’ medical records; indeed, currently does not exist a national influenza vaccination registry, and GP captured influenza vaccination data through a process in which data were initially recorded on paper and subsequently manually entered into a database; therefore, if some individuals were vaccinated outside GP practices these were considered unvaccinated. However, being the elderly individuals vaccinated free of charge, the proportion of those who paid out-of-pocket for influenza vaccine is likely to be negligible. All cases did not receive a laboratory-confirmed diagnosis of influenza; moreover, since specific causes of death were not available for all patients, only death for all-causes was considered as outcome. Since the exact vaccination date was not always available, it was not possible to define vaccinated as those subjects with an influenza vaccine administration >14 days before outcome onset. Finally, other possible factors associated with the evaluated outcomes, not considered in this analysis, could have affected the estimated IVE.

## Conclusions

Study results suggest that vaccination remains the most effective preventive measure against severe influenza, among elderly people. Our findings confirm that influenza vaccination is associated with a lower risk of influenza-related complications, indicating the benefit of flu vaccination in the elderly. Further strategies should be considered for improving influenza vaccine coverage, especially among subgroups with a higher risk of more serious events. Therefore, efforts should be focused on improvement of the immunization programs as important instrument of prevention.
