# Supplementary material for: Determinants of vaccination uptake, and influenza vaccine effectiveness in preventing deaths and hospital admissions in the elderly population; Treviso, Italy, 2014/2015-2016/2017 seasons
Source: Hum Vaccin Immunother. 2019 Oct 7;16(2):301–12. doi: 10.1080/21645515.2019.1661754 (PMC7062427; doi:10.1080/21645515.2019.1661754)
Supplement: Supplemental Material [file khvi-16-02-1661754-s001.zip › Supplementary Material.docx]

**Supplementary Material**

**Tables**

**Table S1.** ICD-9-CM codes for identifying influenza-related hospitalizations

**Table S2**. Crude and adjusted incidence rate ratios of being vaccinated by year, LHU 9, Treviso, Veneto Region, Italy 2014-2016

**Table S3.** Incidence rate ratios of being vaccinated, LHU 9, Treviso, Veneto Region, Italy 2014-2016

**Table S4.** Crude incidence rates of death and hospitalization, LHU 9, Treviso, Veneto Region, Italy 2014-2017

**Table S5.** Incidence rate ratios of deaths and influenza-related hospitalizations during 2016/17 influenza season, LHU, Treviso, Veneto Region, Italy

**Figures**

**Figure S1.** Study flow-chart

**Figure S2.** Percentage of vaccinated patients (65 years old or more) (y-axis) by general practitioner (x-axis). General practitioners are ordered from the lowest to the highest percentage.
