# Supplementary material for: Determinants of vaccination uptake, and influenza vaccine effectiveness in preventing deaths and hospital admissions in the elderly population; Treviso, Italy, 2014/2015-2016/2017 seasons
Source: Hum Vaccin Immunother. 2019 Oct 7;16(2):301–12. doi: 10.1080/21645515.2019.1661754 (PMC7062427; doi:10.1080/21645515.2019.1661754)
Supplement: Supplemental Material [file khvi-16-02-1661754-s001.zip › Supplementary Tables S1-S5.docx]

**Table S1.** ICD-9-CM codes for identifying influenza-related hospitalizations

| **ICD-9-CM Diagnosis code** | **Description** |
| --- | --- |
| 487 | Influenza |
| 480-486 | Pneumonia |
| 460-466, 490-496, 500-508, 510-516 | Respiratory diseases |
| 410, 422, 427, 428 | Cardiovascular diseases |
| **487** | **Influenza** |
| 487.0 | With pneumonia |
| 487.1 | With other respiratory manifestations |
| 487.8 | With other manifestations |
| **480** | **Viral pneumonia** |
| 480.0 | Pneumonia due to adenovirus |
| 480.1 | Pneumonia due to respiratory syncytial virus |
| 480.2 | Pneumonia due to parainfluenza virus |
| 480.8 | Pneumonia due to other virus not elsewhere classified |
| 480.9 | Viral pneumonia, unspecified |
| **481** | **Pneumococcal pneumonia** |
| **482** | **Other bacterial pneumonia** |
| 482.0 | Pneumonia due to Klebsiella pneumoniae |
| 482.1 | Pneumonia due to Pseudomonas |
| 482.2 | Pneumonia due to Hemophilus influenzae |
| 482.3 | Pneumonia due to Streptococcus |
| 482.30 | Streptococcus, unspecified |
| 482.31 | Group A |
| 482.32 | Group B |
| 482.39 | Other Streptococcus |
| 482.4 | Pneumonia due to Staphylococcus |
| 482.8 | Pneumonia due to other specified bacteria |
| 482.81 | Anaerobes |
| 482.82 | Escherichia coli |
| 482.83 | Other gram-negative bacteria |
| 482.89 | Other specified bacteria |
| 482.9 | Bacterial pneumonia unspecified |
| **483** | **Pneumonia due to other specified organism** |
| 483.0 | Mycoplasma pneumoniae |
| 483.1 | Chlamydia |
| 483.8 | Other specified organism |
| **484** | **Pneumonia in infectious diseases classified elsewhere** |
| 484.1 | Pneumonia in cytomegalic inclusion disease |
| 484.3 | Pneumonia in whooping cough |
| 484.5 | Pneumonia in anthrax |
| 484.6 | Pneumonia in aspergillosis |
| 484.7 | Pneumonia in other systemic mycoses |
| 484.8 | Pneumonia in other infectious diseases classified elsewhere |
| **485** | **Bronchopneumonia, organism unspecified** |
| **486** | **Pneumonia, organism unspecified** |
| **460** | **Acute nasopharyngitis** |
| **461** | **Acute sinusitis** |
| 461.0 | Maxillary |
| 461.1 | Frontal |
| 461.2 | Ethmoidal |
| 461.3 | Sphenoidal |
| 461.8 | Other acute sinusitis |
| 461.9 | Acute sinusitis, unspecified |
| **462** | **Acute pharyngitis** |
| **463** | **Acute tonsillitis** |
| **464** | **Acute laryngitis and tracheitis** |
| 464.0 | Acute laryngitis |
| 464.1 | Acute tracheitis |
| 464.10 | Without mention of obstruction |
| 464.11 | With obstruction |
| 464.2 | Acute laryngotracheitis |
| 464.20 | Without mention of obstruction |
| 464.21 | With obstruction |
| 464.3 | Acute epiglottitis |
| 464.30 | Without mention of obstruction |
| 464.31 | With obstruction |
| 464.4 | Croup |
| **465** | **Acute upper respiratory infections of multiple or unspecified** |
| 465.0 | Acute laryngopharyngitis |
| 465.8 | Other multiple sites |
| 465.9 | Unspecified site |
| **466** | **Acute bronchitis and bronchiolitis** |
| 466.0 | Acute bronchitis |
| 466.1 | Acute bronchiolitis |
| 466.11 | Acute bronchiolitis due to respiratory syncytial virus (RSV) |
| 466.19 | Acute bronchiolitis due to other infectious organisms |
| **490** | **Bronchitis, not specified as acute or chronic** |
| **491** | **Chronic bronchitis** |
| 491.0 | Simple chronic bronchitis |
| 491.1 | Mucopurulent chronic bronchitis |
| 491.2 | Obstructive chronic bronchitis |
| 491.20 | Without mention of acute exacerbation |
| 491.21 | With acute exacerbation |
| 491.8 | Other chronic bronchitis |
| 491.9 | Unspecified chronic bronchitis |
| **492** | **Emphysema** |
| 492.0 | Emphysematous bleb |
| 492.8 | Other emphysema |
| **493** | **Asthma** |
| 493.0 | Extrinsic asthma |
| 493.1 | Intrinsic asthma |
| 493.2 | Chronic obstructive asthma |
| 493.9 | Asthma, unspecified |
| **494** | **Bronchiectasis** |
| **495** | **Extrinsic allergic alveolitis** |
| 495.0 | Farmers' lung |
| 495.1 | Bagassosis |
| 495.2 | Bird-fanciers' lung |
| 495.3 | Suberosis |
| 495.4 | Malt workers' lung |
| 495.5 | Mushroom workers' lung |
| 495.6 | Maple bark-strippers' lung |
| 495.7 | Ventilation pneumonitis |
| 495.8 | Other specified allergic alveolitis and pneumonitis |
| 495.9 | Unspecified allergic alveolitis and pneumonitis |
| **496** | **Chronic airway obstruction, not elsewhere classified** |
| **500** | **Coal workers' pneumoconiosis** |
| **501** | **Asbestosis** |
| **502** | **Pneumoconiosis due to other silica or silicates** |
| **503** | **Pneumoconiosis due to other inorganic dust** |
| **504** | **Pneumonopathy due to inhalation of other dust** |
| **505** | **Pneumoconiosis, unspecified** |
| **506** | **Respiratory conditions due to chemical fumes and vapors** |
| 506.0 | Bronchitis and pneumonitis due to fumes and vapors |
| 506.1 | Acute pulmonary edema due to fumes and vapors |
| 506.2 | Upper respiratory inflammation due to fumes and vapors |
| 506.3 | Other acute and subacute respiratory conditions due to fumes |
| 506.4 | Chronic respiratory conditions due to fumes and vapors |
| 506.9 | Unspecified respiratory conditions due to fumes and vapors |
| **507** | **Pneumonitis due to solids and liquids** |
| 507.0 | Due to inhalation of food or vomitus |
| 507.1 | Due to inhalation of oils and essences |
| 507.8 | Due to other solids and liquids |
| **508** | **Respiratory conditions due to other and unspecified external** |
| 508.0 | Acute pulmonary manifestations due to radiation |
| 508.1 | Chronic and other pulmonary manifestations due to radiation |
| 508.8 | Respiratory conditions due to other specified external agents |
| 508.9 | Respiratory conditions due to unspecified external agent |
| **510** | **Empyema** |
| 510.0 | With fistula |
| 510.9 | Without mention of fistula |
| **511** | **Pleurisy** |
| 511.0 | Without mention of effusion or current tuberculosis |
| 511.1 | With effusion, with mention of a bacterial cause other than t |
| 511.8 | Other specified forms of effusion, except tuberculous |
| 511.9 | Unspecified pleural effusion |
| **512** | **Pneumothorax** |
| 512.0 | Spontaneous tension pneumothorax |
| 512.1 | Iatrogenic pneumothorax |
| 512.8 | Other spontaneous pneumothorax |
| **513** | **Abscess of lung and mediastinum** |
| 513.0 | Abscess of lung |
| 513.1 | Abscess of mediastinum |
| **514** | **Pulmonary congestion and hypostasis** |
| **515** | **Postinflammatory pulmonary fibrosis** |
| **516** | **Other alveolar and parietoalveolar pneumonopathy** |
| 516.0 | Pulmonary alveolar proteinosis |
| 516.1 | Idiopathic pulmonary hemosiderosis |
| 516.2 | Pulmonary alveolar microlithiasis |
| 516.3 | Idiopathic interstitial pneumonia |
| 516.4 | Lymphangioleiomyomatosis |
| 516.5 | Adult pulmonary Langerhans cell histiocytosis |
| 516.6 | Interstitial lung diseases of childhood |
| 516.8 | Other specified alveolar and parietoalveolar pneumonopathies |
| 516.9 | Unspecified alveolar and parietoalveolar pneumonopathy |
| **410** | **Acute myocardial infarction** |
| 410.0 | Of anterolateral wall |
| 410.1 | Of other anterior wall |
| 410.2 | Of inferolateral wall |
| 410.3 | Of inferoposterior wall |
| 410.4 | Of other inferior wall |
| 410.5 | Of other lateral wall |
| 410.6 | True posterior wall infarction |
| 410.7 | Subendocardial infarction |
| 410.8 | Of other specified sites |
| 410.9 | Unspecified site |
| **422** | **Acute myocarditis** |
| 422.0 | Acute myocarditis in diseases classified elsewhere |
| 422.9 | Other and unspecified acute myocarditis |
| 422.90 | Acute myocarditis, unspecified |
| 422.91 | Idiopathic myocarditis |
| 422.92 | Septic myocarditis |
| 422.93 | Toxic myocarditis |
| 422.99 | Other |
| **427** | **Cardiac dysrhythmias** |
| 427.0 | Paroxysmal supraventricular tachycardia |
| 427.1 | Paroxysmal ventricular tachycardia |
| 427.2 | Paroxysmal tachycardia, unspecified |
| 427.3 | Atrial fibrillation and flutter |
| 427.31 | Atrial fibrillation |
| 427.32 | Atrial flutter |
| 427.4 | Ventricular fibrillation and flutter |
| 427.41 | Ventricular fibrillation |
| 427.42 | Ventricular flutter |
| 427.5 | Cardiac arrest |
| 427.6 | Premature beats |
| 427.60 | Premature beats, unspecified |
| 427.61 | Supraventricular premature beats |
| 427.69 | Other |
| 427.8 | Other specified cardiac dysrhythmias |
| 427.81 | Sinoatrial node dysfunction |
| 427.89 | Other |
| 427.9 | Cardiac dysrhythmia, unspecified |
| **428** | **Heart failure** |
| 428.0 | Congestive heart failure |
| 428.1 | Left heart failure |
| 428.9 | Heart failure, unspecified |

**Table S2**. Crude and adjusted incidence rate ratios of being vaccinated by year, LHU 9, Treviso, Veneto Region, Italy 2014-2016

|  | **2014** | | | |  | **2015** | | | |  | **2016** | | | |
| --- | --- | --- | --- | --- | --- | --- | --- | --- | --- | --- | --- | --- | --- | --- |
| **Variables** | **IRR** | **95% CI** | | ***p-value*** |  | **IRR** | **95% CI** | | ***p-value*** |  | **IRR** | **95% CI** | | ***p-value*** |
| **Univariable analysis** |  |  |  |  |  |  |  |  |  |  |  |  |  |  |
| **Sex** |  |  |  |  |  |  |  |  |  |  |  |  |  |  |
| Female | 1.00 |  |  |  |  | 1.00 |  |  |  |  | 1.00 |  |  |  |
| Male | 1.02 | 1.00 | 1.04 | *0.023* |  | 1.03 | 1.01 | 1.05 | *0.002* |  | 1.05 | 1.03 | 1.06 | *<0.001* |
| **Age group (years)** |  |  |  |  |  |  |  |  |  |  |  |  |  |  |
| 65-69 | 1.00 |  |  |  |  | 1.00 |  |  |  |  | 1.00 |  |  |  |
| 70-74 | 1.53 | 1.48 | 1.57 | *<0.001* |  | 1.51 | 1.46 | 1.55 | *<0.001* |  | 1.41 | 1.37 | 1.45 | *<0.001* |
| 75-79 | 1.90 | 1.85 | 1.96 | *<0.001* |  | 1.87 | 1.81 | 1.93 | *<0.001* |  | 1.74 | 1.69 | 1.79 | *<0.001* |
| 80-84 | 2.09 | 2.02 | 2.16 | *<0.001* |  | 2.06 | 1.99 | 2.13 | *<0.001* |  | 1.91 | 1.85 | 1.97 | *<0.001* |
| 85-89 | 2.15 | 2.07 | 2.23 | *<0.001* |  | 2.15 | 2.08 | 2.23 | *<0.001* |  | 1.98 | 1.91 | 2.05 | *<0.001* |
| ≥90 | 2.20 | 2.10 | 2.30 | *<0.001* |  | 2.14 | 2.05 | 2.24 | *<0.001* |  | 1.97 | 1.89 | 2.06 | *<0.001* |
| **N. of chronic underlying conditions** |  |  |  |  |  |  |  |  |  |  |  |  |  |  |
| 0 | 1.00 |  |  |  |  | 1.00 |  |  |  |  | 1.00 |  |  |  |
| 1 | 1.51 | 1.46 | 1.56 | *<0.001* |  | 1.52 | 1.47 | 1.57 | *<0.001* |  | 1.54 | 1.49 | 1.59 | *<0.001* |
| 2 | 1.81 | 1.75 | 1.87 | *<0.001* |  | 1.86 | 1.80 | 1.92 | *<0.001* |  | 1.86 | 1.80 | 1.92 | *<0.001* |
| 3 | 2.06 | 1.99 | 2.14 | *<0.001* |  | 2.09 | 2.01 | 2.16 | *<0.001* |  | 2.07 | 2.00 | 2.15 | *<0.001* |
| >3 | 2.27 | 2.18 | 2.35 | *<0.001* |  | 2.30 | 2.22 | 2.39 | *<0.001* |  | 2.28 | 2.20 | 2.37 | *<0.001* |
| **Pneumococcal vaccination** | 0.79 | 0.77 | 0.81 | *<0.001* |  | 0.82 | 0.79 | 0.84 | *<0.001* |  | 0.87 | 0.84 | 0.89 | *<0.001* |
| **Expenses for specialist medical care (Euros)^1^** |  |  |  |  |  |  |  |  |  |  |  |  |  |  |
| < 300 | 1.00 |  |  |  |  | 1.00 |  |  |  |  | 1.00 |  |  |  |
| 300-700 | 1.59 | 1.54 | 1.64 | *<0.001* |  | 1.60 | 1.55 | 1.65 | *<0.001* |  | 1.56 | 1.52 | 1.61 | *<0.001* |
| 700-1,700 | 1.91 | 1.86 | 1.97 | *<0.001* |  | 1.93 | 1.87 | 1.99 | *<0.001* |  | 1.84 | 1.79 | 1.89 | *<0.001* |
| ≥ 1,700 | 1.93 | 1.88 | 1.99 | *<0.001* |  | 1.92 | 1.87 | 1.98 | *<0.001* |  | 1.83 | 1.78 | 1.88 | *<0.001* |
| **Multivariable analysis** |  |  |  |  |  |  |  |  |  |  |  |  |  |  |
| **Sex** |  |  |  |  |  |  |  |  |  |  |  |  |  |  |
| Female | 1.00 |  |  |  |  | 1.00 |  |  |  |  | 1.00 |  |  |  |
| Male | 1.05 | 1.03 | 1.07 | *<0.001* |  | 1.05 | 1.03 | 1.08 | *<0.001* |  | 1.06 | 1.04 | 1.08 | *<0.001* |
| **Age group (years)** |  |  |  |  |  |  |  |  |  |  |  |  |  |  |
| 65-69 | 1.00 |  |  |  |  | 1.00 |  |  |  |  | 1.00 |  |  |  |
| 70-74 | 1.99 | 1.90 | 2.07 | *<0.001* |  | 1.93 | 1.85 | 2.01 | *<0.001* |  | 1.72 | 1.65 | 1.78 | *<0.001* |
| 75-79 | 2.38 | 2.28 | 2.49 | *<0.001* |  | 2.32 | 2.23 | 2.42 | *<0.001* |  | 2.05 | 1.98 | 2.14 | *<0.001* |
| 80-84 | 2.55 | 2.44 | 2.67 | *<0.001* |  | 2.49 | 2.38 | 2.60 | *<0.001* |  | 2.19 | 2.11 | 2.28 | *<0.001* |
| 85-89 | 2.63 | 2.51 | 2.76 | *<0.001* |  | 2.60 | 2.48 | 2.72 | *<0.001* |  | 2.27 | 2.17 | 2.37 | *<0.001* |
| ≥ 90 | 2.73 | 2.58 | 2.88 | *<0.001* |  | 2.61 | 2.48 | 2.75 | *<0.001* |  | 2.30 | 2.19 | 2.42 | *<0.001* |
| **N. of chronic underlying conditions** |  |  |  |  |  |  |  |  |  |  |  |  |  |  |
| 0 |  |  |  |  |  |  |  |  |  |  |  |  |  |  |
| 1 | 1.25 | 1.21 | 1.29 | *<0.001* |  | 1.27 | 1.22 | 1.31 | *<0.001* |  | 1.29 | 1.25 | 1.34 | *<0.001* |
| 2 | 1.34 | 1.30 | 1.39 | *<0.001* |  | 1.38 | 1.33 | 1.43 | *<0.001* |  | 1.42 | 1.37 | 1.47 | *<0.001* |
| 3 | 1.44 | 1.39 | 1.50 | *<0.001* |  | 1.46 | 1.41 | 1.52 | *<0.001* |  | 1.50 | 1.44 | 1.56 | *<0.001* |
| >3 | 1.51 | 1.44 | 1.57 | *<0.001* |  | 1.53 | 1.47 | 1.60 | *<0.001* |  | 1.58 | 1.51 | 1.64 | *<0.001* |
| **Pneumococcal vaccination** | 1.73 | 1.65 | 1.81 | *<0.001* |  | 1.72 | 1.65 | 1.79 | *<0.001* |  | 1.62 | 1.56 | 1.68 | *<0.001* |
| **Expenses for specialist medical care (Euros)** |  |  |  |  |  |  |  |  |  |  |  |  |  |  |
| <300 | 1.00 |  |  |  |  | 1.00 |  |  |  |  | 1.00 |  |  |  |
| 300-700 | 1.33 | 1.29 | 1.38 | *<0.001* |  | 1.33 | 1.29 | 1.37 | *<0.001* |  | 1.31 | 1.27 | 1.35 | *<0.001* |
| 700-1,700 | 1.47 | 1.43 | 1.52 | *<0.001* |  | 1.48 | 1.43 | 1.53 | *<0.001* |  | 1.42 | 1.38 | 1.46 | *<0.001* |
| ≥1,700 | 1.42 | 1.37 | 1.47 | *<0.001* |  | 1.41 | 1.36 | 1.46 | *<0.001* |  | 1.37 | 1.33 | 1.41 | *<0.001* |

Expenses for specialist medical care: total amount spent in the 12 months before the start of the vaccination campaign. CI, Confidence interval. IRR (Incidence Rate Ratios) were obtained from a multilevel Poisson model where people were clustered by general practitioner. General practitioner was significantly associated in the analysis (p<0.01, not shown in table).

**Table S3.** Incidence rate ratios of being vaccinated, LHU 9, Treviso, Veneto Region, Italy 2014-2016

| **Variables** | **IRR** | **95% CI** | | ***p-value*** |
| --- | --- | --- | --- | --- |
| **Sex** |  |  |  |  |
| Female | 1.00 |  |  |  |
| Male | 1.06 | 1.04 | 1.07 | *<0.001* |
| **Age group (years)** |  |  |  |  |
| 65-69 | 1.00 |  |  |  |
| 70-74 | 1.86 | 1.82 | 1.91 | *<0.001* |
| 75-79 | 2.24 | 2.18 | 2.29 | *<0.001* |
| 80-84 | 2.39 | 2.33 | 2.45 | *<0.001* |
| 85-89 | 2.48 | 2.42 | 2.55 | *<0.001* |
| ≥90 | 2.52 | 2.45 | 2.60 | *<0.001* |
| **N. of chronic underlying conditions** |  |  |  |  |
| 0 | 1.00 |  |  |  |
| 1 | 1.27 | 1.24 | 1.29 | *<0.001* |
| 2 | 1.38 | 1.35 | 1.41 | *<0.001* |
| 3 | 1.47 | 1.44 | 1.50 | *<0.001* |
| >3 | 1.54 | 1.50 | 1.57 | *<0.001* |
| **Pneumococcal vaccination** | 1.68 | 1.64 | 1.72 | *<0.001* |
| **Expenses for specialist medical care (Euros)** |  |  |  |  |
| < 300 | 1.00 |  |  |  |
| 300-700 | 1.32 | 1.30 | 1.35 | *<0.001* |
| 700-1,700 | 1.45 | 1.43 | 1.48 | *<0.001* |
| ≥ 1,700 | 1.40 | 1.37 | 1.43 | *<0.001* |
| **Calendar year** |  |  |  |  |
| 2014 | 1.00 |  |  |  |
| 2015 | 1.00 | 0.99 | 1.01 | *0.870* |
| 2016 | 1.03 | 1.02 | 1.05 | *<0.001* |

Expenses for specialist medical care: total amount spent in the 12 months before the start of the vaccination campaign. CI, Confidence Interval. IRR (Incidence Rate Ratios) were obtained from a multilevel Poisson model where people were clustered by general practitioner. General practitioner was significantly associated in the analysis (p<0.01, not shown in table).

**Table S4.** Crude incidence rates of death and hospitalization, LHU 9, Treviso, Veneto Region, Italy 2014-2017

| **Influenza vaccine** | **N. of events** | **Person-years** | **IR x 1000** | **95% CI** |
| --- | --- | --- | --- | --- |
| **Deaths (all causes)** |  |  |  |  |
| Vaccinated | 4,855 | 125,253 | 38.8 | 37.7 - 39.9 |
| Unvaccinated | 3,276 | 123,752 | 26.5 | 25.6 - 27.4 |
| **Hospitalizations** |  |  |  |  |
| **All causes** |  |  |  |  |
| Vaccinated | 32,612 | 125,253 | 260.4 | 257.6 - 263.2 |
| Unvaccinated | 23,714 | 123,752 | 191.6 | 189.2 - 194.1 |
| **Influenza-related** |  |  |  |  |
| Vaccinated | 11,712 | 125,253 | 93.5 | 91.8 - 95.2 |
| Unvaccinated | 6,643 | 123,752 | 53.7 | 52.4 - 55.0 |
| **Influenza** |  |  |  |  |
| Vaccinated | 177 | 125,253 | 1.4 | 1.2 - 1.6 |
| Unvaccinated | 152 | 123,752 | 1.2 | 1.0 - 1.4 |
| **Pneumonia** |  |  |  |  |
| Vaccinated | 3,175 | 125,253 | 25.3 | 24.5 - 26.2 |
| Unvaccinated | 1,839 | 123,752 | 14.9 | 14.2 - 15.5 |
| **Respiratory diseases** |  |  |  |  |
| Vaccinated | 3,205 | 125,253 | 25.6 | 24.7 - 26.5 |
| Unvaccinated | 1,685 | 123,752 | 13.6 | 13.0 - 14.3 |
| **Cardiovascular diseases** |  |  |  |  |
| Vaccinated | 7,837 | 125,253 | 62.6 | 61.2 - 64.0 |
| Unvaccinated | 4,276 | 123,752 | 34.5 | 33.5 - 35.6 |

IR, Incidence Rate; CI, Confidence Interval.

**Table S5.** Incidence rate ratios of death and influenza-related hospitalizations during 2016/17 influenza season, LHU 9, Treviso, Veneto Region, Italy

|  | **Deaths 2016/17** | | | | **Influenza-related hospitalizations 2016/17** | | | |
| --- | --- | --- | --- | --- | --- | --- | --- | --- |
| **Variables** | **IRR** | **95% CI** | | ***p-value*** | **IRR** | **95% CI** | | ***p-value*** |
| **Vaccinated vs Unvaccinated 2016** |  |  |  |  |  |  |  |  |
| No previous influenza vaccination | 0.95 | 0.65 | 1.39 | *0.781* | 1.08 | 0.87 | 1.35 | 0.467 |
| Influenza vaccination in any of the previous two seasons | 0.34 | 0.27 | 0.43 | *<0.001* | 0.67 | 0.57 | 0.79 | *<0.001* |
| **Sex** |  |  |  |  |  |  |  |  |
| Female | 1.00 |  |  |  | 1.00 |  |  |  |
| Male | 1.33 | 1.13 | 1.56 | *0.001* | 1.32 | 1.20 | 1.45 | *<0.001* |
| **Age group (years)** |  |  |  |  |  |  |  |  |
| 65-69 | 1.00 |  |  |  | 1.00 |  |  |  |
| 70-74 | 1.00 | 0.60 | 1.67 | *1.000* | 1.56 | 1.17 | 2.07 | *0.002* |
| 75-79 | 1.90 | 1.16 | 3.11 | *0.011* | 2.06 | 1.55 | 2.74 | *<0.001* |
| 80-84 | 3.73 | 2.29 | 6.09 | *<0.001* | 3.38 | 2.54 | 4.51 | *<0.001* |
| 85-89 | 7.06 | 4.31 | 11.56 | *<0.001* | 5.34 | 4.00 | 7.15 | *<0.001* |
| ≥90 | 22.07 | 13.50 | 36.08 | *<0.001* | 7.63 | 5.67 | 10.26 | *<0.001* |
| **N. of Clinical conditions** |  |  |  |  |  |  |  |  |
| 0 | 1.00 |  |  |  | 1.00 |  |  |  |
| 1 | 1.07 | 0.75 | 1.52 | *0.706* | 1.20 | 0.96 | 1.51 | *0.112* |
| 2 | 1.30 | 0.92 | 1.86 | *0.141* | 1.46 | 1.16 | 1.84 | *0.001* |
| 3 | 1.98 | 1.39 | 2.83 | *<0.001* | 2.47 | 1.96 | 3.11 | *<0.001* |
| >3 | 2.86 | 2.00 | 4.09 | *<0.001* | 4.42 | 3.52 | 5.56 | *<0.001* |
| **Pneumococcal vaccination** | 0.93 | 0.56 | 1.55 | *0.778* | 1.10 | 0.83 | 1.46 | 0.515 |
| **Expenses for specialist medical care (Euros)** |  |  |  |  |  |  |  |  |
| < 300 | 1.00 |  |  |  | 1.00 |  |  |  |
| 300-700 | 0.81 | 0.58 | 1.13 | *0.205* | 1.01 | 0.81 | 1.25 | 0.938 |
| 700-1,700 | 0.89 | 0.65 | 1.24 | *0.500* | 1.33 | 1.09 | 1.63 | 0.005 |
| ≥ 1,700 | 4.14 | 3.12 | 5.50 | *<0.001* | 3.32 | 2.75 | 4.01 | *<0.001* |

Expenses for specialist medical care: total amount spent in the 12 months before the start of the vaccination campaign. CI, Confidence Interval. IRR (Incidence Rate Ratios) were obtained from a Poisson regression model.
